# Adipokines and their role in acute pancreatitis

**DOI:** 10.5937/jomb0-47515

**Published:** 2024-06-15

**Authors:** Saira Rafaqat, Irena Radoman-Vujačić, Dimitrios Patoulias, Huma Khurshid, Aleksandra Klisić

**Affiliations:** 1 Lahore College for Women University, Department of Zoology, Lahore, Punjab, Pakistan; 2 University of Montenegro, Faculty of Medicine, Clinical Center of Montenegro, Department of Internal Medicine, Podgorica, Montenegro; 3 Aristotle University of Thessaloniki, General Hospital "Hippokration", Second Department of Cardiology, Outpatient Department of Cardiometabolic Medicine, Thessaloniki, Greece; 4 University of Montenegro, Faculty of Medicine, Podgorica, Montenegro; 5 Primary Health Care Center, Center for Laboratory Diagnostics, Podgorica, Montenegro

**Keywords:** acute pancreatitis, adipokines, adipose tissue, obesity, akutni pankreatitis, adipokini, masno tkivo, gojaznost

## Abstract

Acute pancreatitis (AP) is characterized by an inflammatory response that leads to edema and haemorrhaging of pancreatic tissue. In severe cases, it can even result in the necrosis of pancreatic tissue following activation within the pancreas. Adipokines are biologically active molecules released by adipose tissue that have a wide-ranging impact on health and disease. Adipokines are cytokines produced not only in white adipose tissue but also in the fat surrounding the pancreas, and they play a role in the body's inflammatory response. The presence of increased adipose tissue, often associated with obesity, has been linked to a heightened systemic inflammatory response in cases of AP. According to the literature, there are many adipokines. This article summarizes the role of adipokines in AP. Adipokines could be promising biomarkers for both diagnostic and new therapeutic treatment strategies in AP. However, a deeper knowledge of the signaling pathways of adipokines and their potential therapeutic role in AP is necessary.

## Abstract (sr)

Akutni pankreatitis (AP) se karakteriše inflamatornim odgovorom koji vodi edemu i hemoragiji tkiva pankreasa. U težim stanjima može voditi i nekrozi pankreasa. Adipokini su biološki aktivni molekuli koje sekretuje masno tkivo, a koji imaju širok spektar dejstva. Adipokini i citokini nisu samo produkt belog masnog tkiva, već i masnog tkiva koje okružuje pankreas, igrajući važnu ulogu u inflamatornom odgovoru organizma. Prisustvo uvećanog masnog tkiva, često povezano sa gojaznošću, dovodi se u vezu sa pojačanim inflamatornim odgovorom u slučajevima AP. Podaci iz literature ukazuju na veliki broj otkrivenih adipokina. Ovaj revijski članak predstavlja prikaz uloge pojedinih adipokina u AP. Adipokini mogu biti obećavajući dijagnostički i terapijski biomarkeri u AP. Ipak, potrebna su dublja saznanja o signalnim putevima adipokina i potencijalnoj terapijskoj ulozi ovih biomarkera u AP.

## Introduction

Acute pancreatitis (AP) is characterized by an inflammatory response that causes pancreatic tissue to become inflamed, edematous, and hemorrhagic. In extreme situations, the pancreas can become activated, which can lead to the necrosis of pancreatic tissue. Several circumstances can lead to the onset of this illness [1], the development of subcutaneous blood clots, as well as symptoms including stomach discomfort, nausea, vomiting, fever, increased body temperature, jaundice, intestinal obstruction, and evidence of peritoneal irritation. Furthermore, AP can result in side effects such as pancreatic abscesses, pseudocysts, acute respiratory failure, acute renal failure, cardiac issues, pancreatic encephalopathy, sepsis, and fungus infections [2]. Due to conditions including pancreatic hemorrhagic necrosis and organ failure, a portion of AP patients, around 20%–30%, develop severe acute pancreatitis (SAP) [3]. With a 5%–10% mortality rate, SAP requires early discovery and focused treatment to lower death rates [4].

Adipokines are biologically active molecules released by adipose tissue that have a wide-ranging impact on health and disease [5]. They play crucial roles in the regulation of metabolism, inflammation, immunity, cardiovascular function, and even cancer. However, when adipokines become dysregulated, they can contribute to disorders associated with obesity [5]. It is important to note that adipose tissue is not merely a passive storage site for energy but also functions as an active endocrine organ, releasing various molecules known as adipokines [6]. Adipokines are a class of cytokines that exert regulatory control over processes such as inflammation, metabolism, appetite, cardiovascular function, and immune responses. Common examples of adipokines include leptin, adiponectin, and resistin, among others. The specific type and quantity of adipokines produced by adipose tissue depend on factors like the type of adipocytes (white or brown), their size, number, location within the body and interactions with other cells [5]. Adipocytes, or fat cells [6], can be categorized into two primary types: white adipocytes, which store excess energy as triglycerides and release various adipocytokines like leptin, adipsin, adiponectin, omentin, tumor necrosis factor-alpha (TNF-α), interleukin- 6 (IL-6), monocyte chemoattractant protein-1, plasminogen activator inhibitor-1, resistin, visfatin, and retinol-binding protein 4 (RBP4), and brown adipocytes, which store energy as smaller lipid droplets and secrete cytokines such as fibroblast growth factor-21 (FGF-21), bone morphogenetic protein 7, vascular endothelial growth factor A (VEGF A), irisin, neuregulin 4, nesfatin-1, meteorin-like protein, chemerin, IL-6, IL-8, IL-10. These cytokines have beneficial effects on processes such as thermogenesis, energy expenditure, glucose regulation, lipid metabolism, insulin sensitivity, angiogenesis, and anti-inflammatory responses [6].

Adipokines are produced not only in white adipose tissue, but also in the fat surrounding the pancreas, and they play a role in the overall body's inflammatory response [7]. The presence of increased adipose tissue, often associated with obesity, has been linked to a heightened systemic inflammatory response in cases of AP [7]. Additionally, it can serve as a prognostic factor for outcomes such as mortality, local and systemic complications, and the severity of AP [7]. Peripancreatic fat necrosis in AP has been linked to the emergence of SAP, multiple organ failure, and increased mortality [8] [9]. It is hypothesized that peripancreatic necrosis may lead to a substantial release of adipokines into the bloodstream, making adipokines as potential predictors of the clinical course and complications of AP. Some older studies have reported significant differences in the concentrations of resistin, visfatin, leptin, and adiponectin between patients with mild AP and those with SAP [10] [11] [12] [13] [14] [15] [16]. However, these studies varied widely in their methodologies, diagnostic criteria, classification, and assessment of AP [10] [11] [12] [13] [14] [15] [16].

According to the literature, there are many discovered adipokines so far [5] [6] [7]. In the same way, Yu et al. [17], Karpavicius et al. [18] and Wos-Wroniewicz et al. [19] reported adiponectin, IL-6, resistin, ghrelin, and leptin in AP. This review article summarizes the role of some other adipokines in AP, such as asprosin, apelin, adipsin, angiopoietin-like protein 2 (ANGPTL2), chemerin, FGF-21, progranulin, visfatin, vaspin, omentin-1, RBP4, lipocalin 2, TNF-α, and osteopontin (OPN) since these have not collectively summarized and reported yet.

### Literature search strategy

Science Direct, PubMed, and Google Scholar were among the databases used to perform the literature review. Clinical research was only conducted if it was related to English-language literature and particular keywords like »Acute pancreatitis,« »Adipokines,« and »Pathogenesis« were used. No set timeline was established, even though the emphasis was on current findings. By looking through the reference lists of the chosen papers, additional pertinent publications were found.

### Adipokines in acute pancreatitis

**Figure 1:**
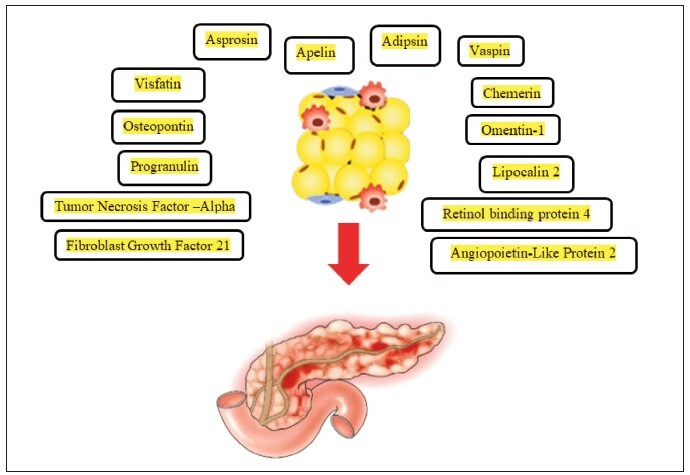
Overall presentation of adipokines in acute pancreatitis.

As already mentioned, adipokines are signaling molecules secreted by adipose tissue (i.e., fat cells) and play a significant role in regulating various physiological processes, including inflammation and metabolism [5]. In the context of AP, the role of adipokines has been studied to understand their potential impact on the disease process. The overall presentation of adipokines in AP is explained in Figure 1. Table 1 explains the circulatory levels and pathophysiological aspects of adipokines in AP. The summary of the studies which explain the adipokines in AP is presented in Table 2.

**Table 1:** The circulating levels and pathophysiological aspects of adipokines in acute pancreatitis. (↑)-increased levels, (?)-no studies have been reported yet, (↕)-not sure. Angiopoietin-like protein 2 (ANGPTL2), Acute pancreatitis (AP), Fibroblast growth factor-21 (FGF-21), Tumor necrosis factor -alpha (TNF-α)

Adipokines	Acute pancreatitis	
	Circulating <br>levels	Pathophysiological aspects
ANGPTL2	?	Undetermined
Chemerin	↕	Chemerin notably alleviated the severity of AP in rats, potentially by inhibiting pro-inflammatory signalling in pancreatic cells (25)
FGF-21	↑	FGF-21 has shown the ability to mitigate pancreatic fibrosis and inflammation in AP ﻿(30). <br>The administration of FGF-21 effectively reverses cerulein-induced pancreatic damage and mitigates autophagy-related harm in both *in vivo* and *in vitro* settings. The mechanism underlying this involves FGF-21 acting on pancreatic acinar cells to stimulate the expression of Sirtuin-1 (Sirt1), which subsequently restores impaired autophagy processes and enables repair damaged organs. Furthermore, when Sirt1 was blocked, cerulein-induced pancreatic injury accelerated, and the regulatory impact of FGF-21 on autophagy activation in mice was diminished. These findings shows that FGF-21 shields against cerulein-induced AP by activating the Sirtuin-1-autophagy axis (30).
Osteopontin	↑	Undetermined
Visfatin	↑	Patients with higher scores for pancreatic and extrapancreatic necrosis had higher visfatin levels upon admission (13).
TNF-α	↑	Degradation and inactivation of TNF-α by pancreatic proteases implies that TNF-α is unlikely to play a key role in the of distant organ failure onset and progression (50).TNF-α exhibits a role in the progression of AP by taking part in regulation of apoptosis (53).TNF-α jointly triggers both proapoptotic and antiapoptotic mechanisms within pancreatic AR4-2J cells (53).TNF-α might lead to transition of AP from a localized condition to a systemic disorder (55).
RBP4	↑	Synthetic derivative of retinoic acid, i.e. fenretinide ameliorates glucose tolerance and insulin sensitivity (58), thus pointing out that the medications that lower RBP4 exhibit metabolically favorable properties. It is presumed that fenretinide activates retinoic acid receptors/retinoid- X receptors pathways in tissues that exhibit active metabolic processes, such as liver and adipose tissue (58).
LCN-2	↑	Inhibitors of expression of LCN-2, i.e., thiazolidinediones (e.g., rosiglitazone and pioglitazone) exhibit anti-inflammatory and insulin-sensitizing properties and show promising therapeutic potential in lowering LCN-2 (72).
Vaspin	↕	The anti-inflammatory properties of vaspin have been atributed to its capability for inhibition of the expression of proinflammatory adhesion molecules, such as E-selectin, vascular cell adhesion molecule 1 (VCAM-1), intercellular adhesion molecule 1 (ICAM-1), and monocyte chemoattractant protein 1 (MCP-1) via inhibition of NF-kB signaling pathways (74, 75). The anti-inflammatory and insulin-sensitizing properties of vaspin are assumed to be a compensatory process due to insulin resistant state (73).
Omentin-1	↑	Anti-inflammatory, anti-atherogenic and insulin-sensitizing properties (73, 79) make omentin- 1 as a promising biomarker for future trials of tissue-specific insulin resistance.
Asprosin	?	Undetermined
Adipsin	?	Undetermined
Apelin	↑	The exposure of this peptide can exhibit a protective impact in chronic pancreatitis, by decreasing the inflammation and the stage of fibrosis and cells degeneration in pancreas (89).
Progranulin	?	Undetermined

**Table 2:** Summary of the studies which explain the adipokines in acute pancreatitis.

First author	Year	Adipokines	The main finding in acute pancreatitis	ref
Lv et al.	2021	ANGPTL2	It was revealed that ANGPTL2 has shown excellent diagnostic accuracy for identifying organ failure in AP patients, with combined sensitivity and specificity values of 0.93 and 0.85, respectively.	24
Jaworek et al.	2019	Chemerin	It was suggested that the anti-inflammatory effect of chemerin might be attributed to the attenuation of NF-kB signalling. Chemerin notably alleviated the severity of AP in rats, potentially by inhibiting pro-inflammatory signalling in pancreatic cells.	25
Chen et al.	2020	FGF-21	Levels of FGF-21 in the bloodstream significantly rise in both AP patients and mice with cerulein-induced AP, which correlates with changes in pancreatic injury.	30
Hernandez et al.	2020	FGF-21	The critical role of FGF-21 in safeguarding the function of the exocrine pancreas and suggesting its potential therapeutic target in preventing and treating pancreatitis.	31
Shenoy et al.	2016	FGF-21	Serum FGF-21 levels increased significantly in individuals with AP, implying that the pancreas might contribute to increased FGF-21 levels after injury and FGF-21 may contribute to the recovery process.	32
Wirestam et al.	2023	OPN	OPN was a relevant biomarker reflecting tissue injury in AP. The increase in OPN over time suggests that serial measurements of OPN could aid in the timely recognition of patients at risk.	41
Swärd et al.	2018	OPN	It revealed that OPN levels could be predictors of future organ failure. The relative change in OPN levels between admission and day 2/3 was higher in patients with moderate-to-severe disease.	42
Mao et al.	2020	Visfatin	The serum levels visfatin levels were significantly higher in both, AP and SAP groups as compared to the control group. Determination of visfatin levels could be a key marker for the early diagnosis of SAP and the assessment of patients' prognoses.	45
Schäffler et al.	2010	Visfatin	Visfatin concentration can function as an early marker for prediction of peripancreatic necrosis and AP clinical severity, thus potentially offering a new and valuable serum diagnostic biomarker in clinical practice.	13
Daniel et al.	2010	Visfatin	Throughout AP, visfatin and resistin levels displayed a joint increase with CRP. This assume that mentioned parameters could potentially serve as additional tool for both the prognosis and monitoring of AP.	46
Alsfasser et al.	2005	TNF-α	It indicated that plasma TNF-α levels might not increase in experimental AP, and in fact, they were notably lower in SAP when compared to sham-operated controls.	50
Grewal et al.	1994	TNF-α	TNF-α might play a major role in the onset of the SAP-systemic complications, and neutralizing TNF-α with a polyclonal antibody might serve as a potential therapeutic approach to alleviate these complications.	51
Hughes et al.	1996	TNF-α	Higher TNF-α levels have been observed in a severe form of AP, and this could play a role in the physiological consequences of the condition.	52
Kıyıcı et al.	2009	TNF-α	TNF-α levels were higher in AP cases, compared to the chronic form of the disease. However, the authors did not find a correlation between TNF-α levels and the disease severity.	54
Osman et al.	1999	TNF-α	It indicated that TNF-α and IL-8 may be a part of driving the transition of AP from a localized form to a systemic disease.	55
Charalabopoulos et al.	2019	TNF-α	There was a significant increase in TNF-α expression over time after surgery in the control group. Conversely, in the apigenin-treated group, there was a significant decrease in TNF-α expression over time after surgery (p<0.013). At the 72-hour mark, apigenin not only that reduced the expression of TNF-α in the pancreas, but it also prevented pancreatic necrosis.	56
Zhang et al.	2017	TNF-α	The induction of heme oxygenase-1 during the early stages of SAP appears to modulate the systemic inflammatory response, protecting against injuries to the pancreas and neighbouring organs, such as the liver, through the inhibition of TNF-α and the increase in IL-10.	57
Han et al.	2023	RBP4	Significant predictive ability of RBP4 for severity of AP and related complications, i.e., acute lung injury (AUC=0.829) and acute necrotic collection (AUC=0.821) in a study that included nearly 1.900 patients with AP.	63
Singh et al.	2017	LCN-2	Significant correlation between serum LCN-2 and WC in total of 92 patients after AP. Alcohol consumption can additionally aggravate the dysfunction of adipose tissue.	64
Chakraborty et al.	2010	LCN-2	LCN-2 is proposed as an early biomarker of SAP, being several times higher in SAP as compared to mild AP (MAP) and showing 100% sensitivity and specificity in discriminating SAP from MAP during first 48 hours. A significant relationship of LPN-2 with fatal outcome was also shown.	69
Siddappa	2018	LCN-2	Serum and urine LCN-2 levels were found to be higher in patients with AP on day 1 as compared to controls. Serum and urine LCN-2 concentration can serve as predictor of AP severity and development of its complications, such as AKI (70).	70
Lipinski et al.	2015	LCN-2	Urinary LCN-2 levels on admission and within the first 24 hours in 104 patients with AP and confirmed an increasing trend in AP mortality (AUC=0.980 and 0.920, respectively) and severity (AUC=0.750 and 0.930, respectively) in parallel with increasing urinary LCN-2 levels.	71
Singh et al.	2018	Vaspin	The relationship between central adiposity and several insulin resistance indices (e.g., among them HOMA-IR and HOMA-vaspin) in 92 patients after AP episode was examined. The AUC for HOMA-IR and HOMA-vaspin were 0.698 and 0.735, respectively, showing good concordance between these two insulin resistance indices.	76
Singh et al.	2018	Omentin-1	A good agreement between the AUC for HOMA-IR and HOMA-omentin-1 (AUC=0.698 and 0.756, respectively) was shown in individuals after an AP episode.	76
Sit et al.	2014	Omentin-1	Higher omentin-1 levels were observed in AP rats as compared to controls, as well as in chronic pancreatitis as compared to both, AP and control rats.	81
Han et al.	2017	Apelin	Apelin inhibited the activation of NF-kB which could be the proposed mechanism for diminished values of pro-inflammatory cytokines in pancreas of mice with chronic pancreatitis that were administered apelin. Accordingly, the pancreatic up-regulation of tumor necrosis factor-a, ICAM, IL-1β, were blocked by apelin.	90

### Angiopoietin-like protein 2

Angiopoietin-like protein 2 (ANGPTL2) is primarily synthesized within the visceral fat tissue, where it stimulates the recruitment of macrophages and the formation of new blood vessels [20]. In obese mice, increased ANGPTL2 levels resulted in persistent inflammation and restructuring of adipose tissue, ultimately leading to systemic insulin resistance.

ANGPTL2 is a newly identified adipokine (i.e. it falls under the newly identified ANGPTL family), and it has been linked to various metabolic disorders such as insulin resistance, obesity, and diabetes [20] [21]. Previous research has demonstrated that ANGPTL2 can function as a contributor to persistent inflammation. Despite their structural resemblance to angiopoietin, ANGPTLs do not interact with the conventional angiopoietin receptors. Many members of this family are engaged not only in controlling angiogenesis but also in managing glucose and lipid metabolism [20] [21].

ANGPTL2 is a novel type of glycoprotein that falls within the category of endothelial growth factors [22] [23]. It specifically targets endothelial cells and is stored in secretory Weibel-Palade bodies. When triggered by conditions like hypoxia, inflammation, or mechanical injury, ANGPTL2 is released from endothelial cells [22]. ANGPTL2 plays a role in preparing the endothelium to respond to angiogenic and inflammatory cytokines by binding to the same site on the Tie-2 receptor as angiopoietin 1 [22] [23]. This interaction leads to the destabilization of endothelial cells and vascular degeneration, ultimately causing a vascular leakage syndrome. This syndrome can result in hemoconcentration, hypotension, pulmonary edema, and renal insufficiency in individuals with acute conditions, including AP. Recent research has identified Ang-2 as a promising biomarker for predicting complications, particularly organ failure, associated with AP [22] [23].

Another study was to evaluate the precision of angiopoietin-2 (Ang-2) as a predictive marker for organ failure in AP. It was revealed that Ang-2 exhibits a strong diagnostic accuracy for identifying organ failure in AP patients, with combined sensitivity and specificity values of 0.93 and 0.85, respectively. The area under the receiver operating characteristic curve (AUC) was 0.95, and the diagnostic odds ratio was 83.18. Further subgroup and sensitivity analyses indicated that Ang-2's most effective predictive ability is observed between 24 and 72 hours after the onset of AP. These findings suggest that Ang-2 has the potenpotential to serve as a valuable predictor of AP severity in future clinical applications [24].

### Chemerin

Chemerin works as a potent chemoattractant, attracting cells from the bone marrow and triggering the migration of immune cells like macrophages and natural killer cells to the inflamed location [25]. Chemerin is now understood to be one of the adipokines (adipocytokines), despite once being largely thought to come from adipose tissue. The liver, pancreas, intestinal epithelium, fibroblasts, skin epidermis, platelets, and respiratory tract can also produce chemerin, according to recent studies. There is disagreement on chemerin's contribution to the inflammatory response [25]. Chemerin may either lessen inflammation or perhaps cause it, according to certain studies. Another research looked at chemerin's effects on AP both *in vivo* and *in vitro*. Histological analysis proved the presence of acute pancreatitis. Chemerin treatment reduced AP histological symptoms, serum amylase activity, and TNF-α levels in AP-infected rats [25]. Compared to cultures treated with caerulein alone, TNF-α signalling was reduced in AR42J cells exposed to caerulein and chemerin [25]. It was proposed that the reduction of nuclear factor kappa B (NF-κB) signaling may be responsible for chemerin’s anti-inflammatory effects. Chemerin notably alleviated the severity of AP in rats, potentially by inhibiting pro-inflammatory signalling in pancreatic cells [25].

### Fibroblast growth factor-21

Fibroblast growth factor 21 (FGF-21) belongs to the fibroblast growth factor family and primarily communicates with tissues through a receptor combination comprising fibroblast growth factor-21 receptor (FGFR) and β-klotho [26] [27]. The presence of β-klotho is crucial for enabling FGF-21 to engage with FGFR and initiate the subsequent signalling pathways [26] [27]. FGF-21 has been identified as a metabolic regulator that exerts its effects primarily on the liver, pancreatic islets, and adipose tissue. FGF-21 is considered an adipokine because it is produced and discharged from white adipose tissue. FGF-21 combines several mechanisms to control human energy balance, blood glucose levels, and lipid metabolism [28]. It exhibits complementary functions when compared to adiponectin [29].

Although FGF-21 is notably present in high amounts in the pancreas, its role in AP has remained largely unexplored. Recent findings indicate that FGF-21 expression increases during the progression of AP which mitigates pancreatic fibrosis and inflammation in AP. FGF-21 is a metabolic hormone that has a positive effect on AP and is recognized for its numerous impacts on insulin sensitivity, glucose and lipid metabolism, and other metabolic processes. However, the exact mechanism that underlies this effect is not enlightened yet. Both patients with AP and mice induced to develop AP with cerulein treatment exhibit pathological and physiological changes associated with FGF-21. To understand the mechanisms in volved, researchers examined how FGF-21 affects autophagy in mice treated with FGF-21 and in cultured pancreatic cells [30]. Notably, the blood levels of FGF-21 dramatically increase in AP patients as well as mice that have cerulein-induced AP, and this coincides with alterations in pancreatic damage. The administration of FGF-21 effectively reverses cerulein-induced pancreatic damage and mitigates auto phagy-related harm in both in vivo and in vitro settings. The mechanism behind this involves FGF-21 acting on pancreatic acinar cells to enhance the expression of Sirtuin-1 (Sirt1), which subsequently restores impaired autophagy processes and helps repair damaged organs [30]. Furthermore, when Sirt1 was blocked, cerulein-induced pancreatic injury accelerated, and the regulatory effect of FGF-21 on autophagy activation in mice weakened. These findings illustrate that FGF-21 shields against cerulein-induced AP by activating the Sirtuin-1-autophagy axis [30].

The body's largest quantities of FGF-21, which is essential for maintaining the health of acinar cells, are found in the exocrine pancreas. It has been shown that both acute and chronic pancreatitis in mice and humans results in a reduction in FGF-21 expression as a result of the activation of the integrated stress response (ISR) pathway [31]. When the ISR is activated in cultured acinar cells and mouse pancreata, the scientists looked into the process and found that it causes the synthesis of activating transcription factor 3 (ATF3), a transcriptional repressor. As a result of ATF3 directly attaching to particular areas on the FGF-21 promoter, the expression of FGF-21 is reduced. The fact that the human FGF-21 promoter included these ATF3 binding sites is significant since it suggests a similar regulation mechanism in people. ATF3 and FGF-21 expression patterns in the pancreas of human patients with pancreatitis were found to be consistent with those seen in mice, supporting previous research [31]. These studies showed that the ISR could be successfully countered and pancreatitis was successfully treated by substituting pharmacological techniques for FGF-21. The restoration of FGF-21 expression and relief from pancreatitis was also brought about by suppressing the ISR using an inhibitor of PKR-like endoplasmic reticulum kinase (PERK). These findings highlight the crucial function of FGF-21 in maintaining the exocrine pancreas' functionality and point to its potential therapeutic use in preventing and treating pancreatitis [31].

The acinar pancreas expresses significant quantities of the metabolic regulator FGF-21. However, it is unclear how this protein affects pancreatic functioning. In mouse models of experimental pancreatitis, it appears to have a protective effect. Another study looked into how FGF-21 affects the development and recovery of AP. Serum FGF-21 levels were shown to significantly increase in patients with AP, peaking four to six days after the maximum lipase level and then steadily declining [32]. Peak FGF-21 levels in those with AP were considerably higher than the maximum levels seen in our control group and were also higher than the beginning values. The proportion of active to total FGF-21 remained consistent throughout the disease (42.5% vs. 44.4%, p=0.58). The fold increase in FGF-21 levels was significantly more pronounced in individuals with AP compared to healthy subjects (4.7 vs. 2.0, p=0.01), with even higher increases seen in SAP compared to mild cases (18.2 vs. 4.4, p=0.01). Further, the peak FGF-21 levels coincided with the day when patients were effectively able to restart oral consumption. According to these results, blood FGF-21 levels dramatically rise in people with AP, indicating that the pancreas may contribute to higher FGF-21 levels following damage and may aid in the healing process [32].

### Osteopontin

Osteopontin (OPN), also known as early T lymphocyte activation-1 (Eta-1), uroprotein, and secreted phosphoprotein 1 (SPP-1), is a 314 amino acid long glycophosphoprotein that is strongly phosphorylated [33] [34]. Due to various post-translational modifications, including O-linked glycosylation, phosphorylation, sialylation, and tyrosine sulfation, it has a molecular weight that ranges between 41 and 75 kDa. These alterations can change how the protein reacts in tissues [34] [35]. Osteopontin was originally discovered in bone tissue but exhibits versatile functions due to its widespread presence in major organs and systems [33]. Indeed, OPN has been identified in various anatomical locations, including bone cells, adipocytes, cardiomyocytes, renal cells, and vascular cells [36] [37]. In each of these anatomical compartments, OPN serves as a key player in regulating T-cell activity, macrophage recruitment and infiltration, and the inhibition of local calcification signaling [38] [39]. OPN exists in two main forms: an intracellular form and a soluble form, each mediating both physiological and pathological signalling processes [35] [40].

OPN is also known by various, names such as bone/sialoprotein I, early T-lymphocyte activation-1, and secreted phosphoprotein 1, which is a protein primarily associated with bone remodeling. However, it is released by various cell types, especially during tissue injury and inflammation. OPN can operate as a soluble cytokine in inflamed tissues and the bloodstream and plays a critical role in cell-to-cell and cell-to-matrix interactions essential for the inflammatory response. AP requires a simple-to-measure biomarker to forecast the severity of the condition, as numerous grading systems have been suggested for this goal. The exact function of OPN in AP is unclear, however, it may be released into the circulation quickly in response to tissue damage. Therefore, Wirestam et al. [41] study assessed the plasma OPN levels concerning the severity of AP. Upon admission, patients with AP had significantly higher OPN levels, measuring 156.4 ng/mL (IQR 111.8–196.2 ng/mL), compared to the control group, which had levels of 37.4 ng/mL (IQR 11.7–65.7 ng/mL) (p<0.0001). However, OPN levels at admission could not differentiate between cases of mild and moderate-to-severe disease (132.6 ng/mL vs. 163.4 ng/mL). Nevertheless, it was observed that the changes in OPN within 24 hours of admission and on day 2/3 were more pronounced among patients with moderate-to-severe AP (33.7%) in comparison to those with mild AP (-8.1%) (p=0.01). As a result, it may be inferred that OPN was a pertinent biomarker representing tissue damage in AP. Serial measurements of OPN may help in the early identification of patients at risk, according to the increase in OPN over time. To determine how OPN and AP results relate to one another, prospective studies are required. The results indicate that OPN has the potential as a biomarker for detecting tissue damage in AP, despite the limitations mentioned previously. To determine its therapeutic relevance, more research involving bigger patient populations is necessary [41].

In a more extensive investigation conducted by Swärd et al. [42], involving 86 patients, it was discovered that OPN levels could predict future organ failure. Although, in a group of patients, the initial OPN levels upon admission did not enable us to distinguish between mild and moderate-to-severe cases of the disease, the author observed significantly elevated levels in patients compared to the control group. This suggests the presence of inflammation and/or tissue damage. Additionally, it was noticed a dynamic shift over time, particularly among patients with severe disease, where their OPN levels were notably higher, though this was only statistically significant on day 2. Additionally, individuals with moderate-to-severe illness showed a larger relative shift in OPN levels between day 2/3 and admission. It is crucial to consider the timing of sample collection and the duration of symptoms when assessing a biomarker's ability to predict outcomes [42].

### Visfatin

A polypeptide having a molecular weight of 52 kDa, visfatin contains 491 amino acids [43]. It can attach to the insulin receptor, enabling the breakdown of glucose and encouraging the differentiation, maturation, and accumulation of fat cells. Furthermore, it can activate the NF-kB signaling pathway, promote the generation of inflammatory cells, and contribute to the body's immune system [43].

Visfatin stands out as a significant adipokine discharged by adipose tissue [43] [44]. Its levels notably rise in individuals with obesity, largely due to increased body mass index. In the context of obesity, the adipocytes within adipose tissue undergo both hypertrophy and hyperplasia, leading to the release of various adipocytokines, including visfatin [44]. Notably, visfatin also functions as an enzyme known as nicotinamide phosphoribosyl transferase, making it a prominent adipokine that influences the body's metabolic balance. Visfatin comes in two forms, extracellular and intracellular, and exerts various effects [44]. Both direct and indirect evidence from *in vitro, in vivo*, and clinical studies points to visfatin's role in modulating pathophysiological processes related to obesity and metabolic syndrome. These include heightened inflammation, increased angiogenesis, the synthesis of NAD mononucleotide, and the upregulation of antiapoptotic proteins across various cell types. Visfatin has been connected to several metabolic alterations related to obesity, including diabetes, heart problems, and some forms of cancer [43] [44]. At higher concentrations, visfatin attracts immune cells, leading to chronic inflammation in adipocytes. Furthermore, it induces insulin resistance in numerous tissues and contributes to dysfunction in pancreatic beta cells during later stages [44].

In this particular study, the serum levels of visfatin were notably higher in both the AP and SAP groups when compared to the control group. Furthermore, the SAP group exhibited significantly higher visfatin levels compared to the AP group. Within the SAP group, patients who did not survive had considerably higher levels of visfatin compared to those who survived. As the therapy went on, the serum visfatin levels steadily declined. According to these results, monitoring visfatin levels may be an essential marker for the early detection of SAP and the evaluation of patients' prognoses [45].

Leptin, adiponectin, and resistin are only a few of the adipocytokines that adipocytes in the fat tissue around and inside the pancreas release. Resistin may be able to detect peripancreatic necrosis early on and determine the severity of AP, according to certain reports [13]. If adipocytokine visfatin may potentially serve as a precursor for foretelling peripancreatic necrosis and the severity of the illness, that possibility was looked into by Schäffler et al. [13]. Visfatin levels were shown to be strongly and positively connected with clinical severity, as determined by ratings like the APACHE-II and Ranson scores, as well as clinical outcomes including death and the requirement for medical treatments. At admission, visfatin levels were significantly higher in patients with higher ratings for necrosis in the pancreas and its surroundings. It was shown by receiver operator characteristics that the concentration of visfatin at admission had a positive predictive value of 93.3% for predicting the severity of peripancreatic necrosis. This was determined by using a cutoff value of 1.8 ng/mL and showed an AUC of 0.89, indicating its reliability in this context (p<0.001). Additionally, it exhibited a sensitivity of 93.3%, specificity of 81.8%, likelihood ratio of 5.1, and post-test probability of 93%. As a result, admission visfatin concentration has the potential to be a new and useful serum marker for diagnostic reasons in clinical practice, serving as an early predictive sign for peripancreatic necrosis and the clinical severity of AP [13].

Hormones generated by fat tissue, resistin and visfatin, have pro-inflammatory characteristics. However, there has not been much research done on their role in AP. Resistin and IL-8 serum levels were noticeably higher in AP patients on the day of admission compared to the control group, and they kept increasing on the third and fifth days of hospitalization. Serum visfatin levels in AP patients upon admission were also notably elevated compared to the control group and continued to increase on the third day of hospitalization. By the fifth day, visfatin levels decreased, though they remained higher than the initial admission levels. Correlations were observed between visfatin and resistin levels, as well as between C-reactive protein (CRP) levels and visfatin, resistin, and IL-8 levels. Throughout AP, visfatin and resistin levels displayed a concurrent increase with CRP. This suggests that these parameters could potentially serve as additional tools for both the prognosis and monitoring of AP [46].

### Tumor necrosis factor-alpha

Tumor necrosis factor-alpha (TNF-α) is a protein composed of 157 amino acids arranged in a homotrimer structure, primarily produced by activated macrophages, T-lymphocytes, and natural killer cells [47]. TNF-α is a multifunctional cytokine that is notorious for having negative impacts on several autoimmune and inflammatory illnesses. It is crucial in controlling other proinflammatory cytokines, and adhesion molecules on leukocytes, and it sets the stage for the activation of immune cells. Among the many different inflammatory cytokines, TNF-α is widely recognized. Additionally, it was the first adipokine identified as being generated by adipose tissue, with its production influenced by obesity, and suggested to play a role in metabolic disorders associated with obesity [48].

TNF-α has been implicated in the development of AP in recent research, which has mostly relied on animal models and laboratory investigations. In particular, it helps the organ dysfunction that is frequently present in severe instances and the systemic advancement of the inflammatory response. When determining the severity of AP and identifying patients at risk for consequences such as multiple organ failure and septic shock, healthcare practitioners utilize blood concentrations of TNF-α to measure the substance's levels. TNF-α is most helpful as a predictive tool in the early stages of the disease, but because it is quickly excreted from the bloodstream and its measurement accuracy seems to be time-dependent, this is when the disease first manifests itself. TNF-α has also been researched as a possible pancreatitis treatment target. Despite encouraging laboratory results, it might be difficult to apply these findings in clinical settings, especially given the inconsistent results of sepsis studies. Therefore, the timing of intervention in subsequent clinical trials examining TNF-α neutralization in acute pancreatitis should be closely related to changes in TNF-α serum levels, and inclusion and exclusion criteria should be carefully defined to identify the population most likely to benefit from such treatment [49].

The release of TNF-α is believed to have a significant role in causing systemic effects in acute pancreatitis. However, it was not observed an increase in plasma TNF-α levels in AP, and the authors propose that it may be susceptible to degradation by circulating pancreatic proteases. Plasma TNF-α level was significantly lower in severe pancreatitis compared to sham-operated controls at both 0.5 and 6 hours after the onset of the condition. When exposed to proteases, TNF-α underwent degradation in the presence of trypsin, elastase, and chymotrypsin, with no observed effect when exposed to pepsin. There was a concentration-dependent reduction in the activity of recombinant rat TNF-α (rrTNFα) when pancreatic proteases were present and a complete time-dependent inactivation in the presence of trypsin. In summary, it indicated that plasma TNF-α levels might not increase in experimental AP, and in fact, they were notably lower in SAP when compared to sham-operated controls. Our findings also demonstrate the degradation and inactivation of TNF-α by pancreatic proteases, suggesting that TNF-α was unlikely to play a significant role in the development of distant organ failure [50]. Additionally, TNF-α is an inflammatory cytokine and may be extremely important in the emergence of systemic problems linked to SAP. Another research looked at the possibility of improving some biochemical parameters in a rat model of AP by using a polyclonal antibody to neutralize TNF-α. Rats' bile ducts were injected with synthetic bile to cause pancreatitis. It showed that pretreatment with the polyclonal antibody significantly improved all of the measured parameters when compared to the animals with pancreatitis that received the placebo. Moreover, TNF-α levels, which were elevated in animals with pancreatitis, were significantly reduced in the treated animals. These findings suggest that TNF-α might play a crucial role in the development of the systemic complications associated with SAP, and neutralizing TNF-α with a polyclonal antibody could be a potential therapeutic approach to mitigate these complications [51].

Increased concentrations of TNF-α were detected in a severe form of AP, and this may play a role in the physiological consequences of the condition. In untreated animals with pancreatitis, there was a significant surge in TNF-α activity in the bloodstream during the first three hours after the onset of the disease. However, this TNF-α surge was prevented by prior treatment with an anti-TNF-α antibody. These observations offer a plausible explanation for the improvements seen in various biochemical and histological parameters, as well as overall survival in an experimental rat model [52]. Also, TNF-α plays a role in the progression of AP. Given its involvement in regulating apoptosis, it investigated how TNF-α interacts with the apoptotic pathway in the pancreas. It revealed that TNF-α simultaneously triggers both proapoptotic and antiapoptotic mechanisms within pancreatic AR4-2J cells. The antiapoptotic mechanisms were mediated through NF-κB and mitogenactivated protein (MAP) kinases, and one of the factors responsible for inhibiting apoptosis [53].

In cases of AP, TNF-a levels were elevated compared to the chronic form of the disease. However, the authors did not find a significant correlation between TNF-α concentrations and the severity of the condition. To gain further clarity on this matter, additional research into other inflammatory markers and acute phase reactants alongside TNF-α is warranted [54]. Proinflammatory cytokines such as TNF-α and IL-8 are believed to have a central role in the progression of SAP and the emergence of its systemic complications, notably acute lung injury. It indicated that TNF-α and IL-8 may be involved in driving the transition of AP from a localized condition to a systemic disorder. It is worth considering experimental testing of hydrocortisone following the induction of AP and its clinical application as a preventive measure to mitigate the risk of SAP induced by endoscopic retrograde cholangiopancreatography [55].

The anti-inflammatory effects of apigenin in an experimental model of AP were investigated. The degree of inflammatory response was assessed by examining the tissue expression of the cytokine TNF-α, in addition to histological analysis. In the control group, there was a significant increase in TNF-α expression over time after surgery. Conversely, in the apigenintreated group, there was a significant decrease in TNF-α expression over time after surgery (p<0.013). At the 72-hour mark, apigenin not only reduced the expression of TNF-α in the pancreas, but also prevented pancreatic necrosis. These findings suggest that apigenin can slow down the progression of AP and reduce its severity. As such, apigenin may serve as a complementary component in a more effective therapeutic strategy for the management of AP [56].

Heme oxygenase-1 (HO-1) is a responsive defensive gene with a substantial role in inflammation. HO-1 serves as a protective mechanism for cells and tissues by countering oxidative stress, sustaining microcirculation, and suppressing inflammation. Zhang et al. [57] investigate the impact of HO-1 on the systemic inflammatory response observed in severe acute pancreatitis. In the rat pancreas and liver, SAP significantly triggered the expression of HO-1 mRNA and protein. The administration of hemin notably reduced oxidative stress and TNF-α levels in both plasma and tissues, while increasing the levels of IL-10. Furthermore, the damage inflicted on the pancreas and liver due to SAP was significantly mitigated by hemin treatment. Conversely, the inhibition of HO-1 expression through the administration of Zn-PP exacerbated the injuries caused by severe AP. The induction of HO-1 during the early stages of severe AP appears to modulate the systemic inflammatory response, protecting against injuries to the pancreas and neighbouring organs, such as the liver, through the inhibition of TNF-α and the enhancement of IL-10 [57].

### Retinol binding protein 4 (RBP4)

RBP4 is an adipokine secreted by hepatocytes and belongs to lipocaline family [58]. It is responsible for the transportation of retinol (vitamin A) [58]. However, RBP4 also exhibits many cardiometabolic properties, being associated with obesity, insulin resistance, diabetes mellitus type 2 and related cardiometabolic complications [59] [60] [61] [62].

Han et al. [63] showed a significant predictive ability of RBP4 for severity of AP and related complications, i.e., acute lung injury (AUC=0.829) and acute necrotic collection (AUC=0.821) in a study that included nearly 1.900 patients with AP.

Even though it is regarded that increased abdominal obesity promotes pro-inflammatory milieu in AP patients, RBP4 was not correlated with waist circumference (WC) in two studies conducted by Singh et al. [64] [65], although significant and independent correlation between RBP4 and WC was confirmed in overweight/obese otherwise healthy population [66].

It was shown that a synthetic derivative of retinoic acid, i.e. fenretinide ameliorates glucose tolerance and insulin sensitivity [58], thus pointing out that the medications that lower RBP4 exhibit metabolically favorable properties. It is presumed that fenretinide activates retinoic acid receptors/retinoid-X receptors pathways in tissues that exhibit active metabolic processes, such as liver and adipose tissue [58] These discoveries shed light on treatment modalities for lowering RBP4 in AP.

### Lipocalin-2

Besides RBP4, lipocalin-2 (LCN-2) is another protein that belongs to the lipocalin family which also plays a significant role in obesity-related diseases [64]. LCN-2 can also be found in literature as human neutrophil lipocalin, neutrophil gelatinase-associated lipocalin (NGAL) and siderocalin [67]. Activated neutrophils in an inflammatory state are the source of LCN-2 secretion (67). Consequently, LCN-2 gets linked with siderophores (i.e., bacterial iron-binding protein) which enables prevention of bacterial infections due to its bacteriostatic properties.

Singh et al. [64] showed significant correlation between serum LCN-2 and WC in a total of 92 patients after AP. They also confirmed that alcohol consumption can additionally aggravate the dysfunction of adipose tissue. Another study by Singh et al. [68] confirmed that LCN-2 is an indicator of fatty pancreas, but not fatty liver. The association between LCN-2 and fatty pancreas was independent of central adiposity and insulin resistance [68].

LCN-2 is proposed as an early biomarker of SAP [69], being several times higher in SAP as compared to mild AP (MAP) and showing 100% sensitivity and specificity in discriminating SAP from MAP during the first 48 hours. A significant relationship of LPN-2 with fatal outcome was also shown [69].

Serum and urine LCN-2 levels were found to be higher in patients with AP on day 1 as compared to controls [70]. Serum and urine LCN-2 concentration can serve as predictors of AP severity and development of its complications, such as AKI [70]. Similarly, Lipinski et al. [71] measured urine LCN-2 levels on admission and within the first 24 hours in 104 patients with AP and confirmed an increasing trend in AP mortality (AUC=0.980 and 0.920, respectively) and severity (AUC=0.750 and 0.930, respectively) in parallel with increasing urinary LCN-2 levels.

Inhibitors of expression of LCN-2, i.e., thiazolidinediones (e.g., rosiglitazone and pioglitazone) exhibit anti-inflammatory and insulin-sensitizing properties and show promising therapeutic potential in lowering LCN-2 [72].

### Vaspin

Adipokine vaspin (i.e. visceral adipose tissue-derived serpin protease inhibitor) is a member of the serpin superfamily, secreted by visceral and subcutaneous adipose tissues [73].

The anti-inflammatory properties of vaspin have been attributed to its capability for inhibition of the expression of proinflammatory adhesion molecules, such as E-selectin, vascular cell adhesion molecule 1 (VCAM-1), intercellular adhesion molecule 1 (ICAM-1), and monocyte chemoattractant protein 1 (MCP-1) via inhibition of NF-κB signaling pathways [74] [75].

The anti-inflammatory and insulin-sensitizing properties of vaspin are assumed to be a compensatory process due to insulin resistant state [73]. However, the results in literature are discrepant. Some studies showed higher serum vaspin levels in obesity and diabetes mellitus type 2, whereas the others claim its lower levels in mentioned disorders pointing out the favorable properties in obesity-related disorders [73].

Sing et al. [76] showed that patients with central obesity exhibited higher insulin resistance. The authors explored the relationship between central adiposity and several insulin resistance indices (e.g., among them HOMA-IR and HOMA-vaspin) in 92 patients after AP episode. The AUC for HOMA-IR and HOMA-vaspin were 0.698 and 0.735, respectively, showing good concordance between these two insulin resistance indices [76].

The modulation of the metabolism of lipids is another feature attributed to vaspin properties [77]. The administration of vaspin led to a decrease in free fatty acid concentration and triglycerides and forced the cholesterol efflux in macrophages thanks to its ability to upregulate the ATP-binding cassette transporter A1 (ABCA1) [77]. A recent study has shown that the administration of atorvastatin led to an increase in vaspin, but future investigations for the therapeutic treatment of this adipokine are required [78].

### Omentin-1

Omentin or intelectin is secreted by visceral adipose tissue in two isoforms, i.e. omentin-1 and omentin-2. Omentin-1 is the major isoform in circulation [79]. Lower levels of omentin-1 are shown in obesity-related disorders [73] [80]. Anti-inflammatory, anti-atherogenic and insulin-sensitizing properties [73] [79] make omentin-1 as a promising biomarker for future trials of tissue-specific insulin resistance. In line with this, Sing et al. [76] showed good agreement between the AUC for HOMA-IR and HOMA-omentin-1 (AUC=0.698 and 0.756, respectively) in individuals after an AP episode.

Blood levels of omentin-1 were measured in an animal study that included a rat model of 8 controls, AP, and chronic pancreatitis, respectively [81]. Higher omentin-1 levels were observed in AP rats as compared to controls, as well as in chronic pancreatitis as compared to both, AP and control rats [81].

McKenzie et al. [82] explored the influence of enteral nutrition on adipokines in the AP course and showed an increase in omentin-1 levels due to enteral nutrition. The proposed explanation for such results was the favorable properties of omentin-1 via anti-inflammatory and insulin signaling pathways and, hence, diminishing insulin resistance and better blood glucose control [82]. Similarly as for vaspin, it was also shown that atorvastatin leads to increase in omentin-1 levels in circulation, although the mechanisms that underlie these effects are not well understood [78].

### Asprosin

Asprosin is a relatively novel diabetogenic adipokine secreted mainly by white adipose tissue, and to a lesser extent by pancreatic B-cells, salivary glands, and cartilage. White adipose tissue secretes asprosin during a fasting state [83]. It favours orexigenic and glucogenic properties (i.e. by induction of hepatic glucose production) [83] [84].

The therapeutic ability of asprosin inhibition for the treatment of obesity-related disorders is a promising target. It was recently shown that anti-asprosin monoclonal antibodies lead to decreased appetite, lowering blood glucose, and body weight loss and future researches are needed to enlighten asprosins’ signaling pathways and its receptors [84].

### Adipsin

Adipsin is an adipokine that belongs to the family of serine proteases with a molecular weight of 28 kDa [85]. It is regarded as a biomolecule that maintains the homeostasis of adipose tissue. Another property attributed to adipsin is its ability to increase the secretion of insulin in response to high glucose levels [73] [85]. Furthermore, adipsin has an important role in the catalyzation of C3a, which is an active form of C3 component that leads to consequent enhanced pancreatic insulin secretion [73] [86]. Studies show that familial C3 deficiency is linked with obesity-related diseases, with C3a values being the risk factor for diabetes mellitus type 2 [85]. A few studies reported higher adipsin levels in patients with type 2 diabetes as compared to controls [86] [87]. However, some other human and animal studies showed lower levels of adipsin in type 2 diabetes [85], thus suggesting that this adipokine could increase insulin sensitivity and diminish glucose intolerance in patients with diabetes by its ability to enhance insulin secretion via C3a production. C3a acts on the pancreatic islet cells by increasing C3a receptor and ATP-sensitive K+ channel inhibition, with a consequent increase in intracellular Ca2+ levels [85].

Due to the ability of adipsin to inhibit lipolysis and to favor the transport of glucose for the accumulation of triglycerides in adipose tissue, this adipokine might be a novel therapeutic target in obesity-related disorders [73] [85].

### Apelin

Apelin is a peptide that serves as a ligand of the APJ receptor. Its therapeutic abilities might be favorable for insulin resistance treatment, although in cancer exerts unfavorable effects, by stimulating the tumor growth [88]. Apelin exhibits an impact on exocrine secretion of pancreas, as well as anti-inflammatory activities [89].

The exposure of this peptide can exhibit a protective impact in chronic pancreatitis, by decreasing the inflammation and the stage of fibrosis and cells degeneration in the pancreas [89].

Previous study showed that apelin inhibited the activation of NF-κB which could be the proposed mechanism for diminished values of pro-inflammatory cytokines in the pancreas of mice with chronic pancreatitis that were administered apelin [90]. Accordingly, the pancreatic up-regulation of tumor necrosis factor α, ICAM and IL-1β, were blocked by apelin [90]. These findings proposed new a approach to the treatment of pancreatitis.

### Progranulin

Progranulin (PGRN), also known as granulin-epithelin precursor, proepithelin, or acrogranin is protein mainly expressed in neurons, adipocytes and immune cells [91]. It has several properties in the body, such as cancer growth, tissue remodeling and wound repair [73] [91]. Animal models showed a direct bound of PGRN to tumor necrosis factor receptor (TNFR), thus leading to inhibition of activation of neutrophils and limiting the bond between TNFR and TNF-α. Consequently, this protein can lead to the inhibition of TNF-α-promoted activation of the MAPK and NF-kB and signaling pathways [73].

The role of PGRN in obesity-related disorders has been recently described [73], showing its higher levels in obesity and insulin-resistant state in the majority of studies.

High-fat diet enhanced the expression of PGRN the adipose tissue of rodents [91]. It is related to weight loss and suppression of appetite and weight loss the hypothalamus [92]. However, PGRN exerts similar effects as leptin in obesity. Namely, resistance to this protein in the hypothalamus promotes hyperphagia [92]. The proposed mechanisms of PGRN action are connected with an increase in proopiomelanocortin and a decrease in Agouti-related peptide and neuropeptide Y [92]. Animal models showed that exposure to PGRN inhibited body weight gain, whereas the inhibition of hypothalamic expression of this protein lead to an increase in weight gain [92].

## Conclusion

This article concludes that adipokines play a significant role in the pathogenesis of AP. It is significant to highlight that there is a complicated link between adipokines and AP, and ongoing research is elucidating their specific functions in the illness process. Factors such as the patient's overall health, the timing of adipokines measurements, and the specific adipokines involved can all impact their effects in AP. Adipokines could be the promising biomarkers for both diagnostic and new therapeutic treatment strategies in AP. Therefore, healthcare professionals and researchers need to continue studying this area to develop better strategies for managing and treating AP.

## Dodatak

### Acknowledgement

This work was financially supported in part by a grant from the Ministry of Education, Science and Innovations, Montenegro.

### Financial Support

None.

### Conflict of interest statement

All the authors declare that they have no conflict of interest in this work.
